# A genetic programming approach to development of clinical prediction models: A case study in symptomatic cardiovascular disease

**DOI:** 10.1371/journal.pone.0202685

**Published:** 2018-09-04

**Authors:** Christian A. Bannister, Julian P. Halcox, Craig J. Currie, Alun Preece, Irena Spasić

**Affiliations:** 1 School of Computer Science & Informatics, Cardiff University, Cardiff, United Kingdom; 2 Cochrane Institute of Primary Care & Public Health, School of Medicine, Cardiff University, Cardiff, United Kingdom; 3 Department of Cardiology, Medical School, Swansea University, Swansea, United Kingdom; Politechnika Krakowska im Tadeusza Kosciuszki, POLAND

## Abstract

**Background:**

Genetic programming (GP) is an evolutionary computing methodology capable of identifying complex, non-linear patterns in large data sets. Despite the potential advantages of GP over more typical, frequentist statistical approach methods, its applications to survival analyses are rare, at best. The aim of this study was to determine the utility of GP for the automatic development of clinical prediction models.

**Methods:**

We compared GP against the commonly used Cox regression technique in terms of the development and performance of a cardiovascular risk score using data from the SMART study, a prospective cohort study of patients with symptomatic cardiovascular disease. The composite endpoint was cardiovascular death, non-fatal stroke, and myocardial infarction. A total of 3,873 patients aged 19–82 years were enrolled in the study 1996–2006. The cohort was split 70:30 into derivation and validation sets. The derivation set was used for development of both GP and Cox regression models. These models were then used to predict the discrete hazards at *t* = 1, 3, and 5 years. The predictive ability of both models was evaluated in terms of their risk discrimination and calibration using the validation set.

**Results:**

The discrimination of both models was comparable. At time points *t* = 1, 3, and 5 years the C-index was 0.59, 0.69, 0.64 and 0.66, 0.70, 0.70 for the GP and Cox regression models respectively. At the same time points, the calibration of both models, which was assessed using calibration plots and the generalization of the Hosmer-Lemeshow test statistic, was also comparable, but with the Cox model being better calibrated to the validation data.

**Conclusion:**

Using empirical data, we demonstrated that a prediction model developed automatically by GP has predictive ability comparable to that of manually tuned Cox regression. The GP model was more complex, but it was developed in a fully automated way and comprised fewer covariates. Furthermore, it did not require the expertise normally needed for its derivation, thereby alleviating the knowledge elicitation bottleneck. Overall, GP demonstrated considerable potential as a method for the automated development of clinical prediction models for diagnostic and prognostic purposes.

## Introduction

Prognosis is central to medicine. All diagnostic and therapeutic actions aim to improve prognosis [1]. Physicians and health policy makers need to make predictions on the disease prognosis to support their decision making. Traditionally, clinical predictions have been more implicit in their nature. Today, however, we live in an era of evidence-based medicine, which is defined as "the conscientious, explicit and judicious use of current best evidence in making decisions about the care of individual patients" [2]. Evidence-based medicine applies scientific methods to clinical practice in order to improve the quality of patient care [3]. Patients themselves are involved in their care through shared decision making, where both physicians and patients actively participate in making choices about diagnostic tests and therapeutic interventions [1, 4].

Clinical prediction models may provide the evidence-based input for shared decision making by estimating probabilities of risks and benefits associated with available choices [5]. Clinical prediction models are also referred to as clinical prediction rules, prognostic models, predictive risk models, or risk scores [6]. They combine a variety of characteristics (e.g. features related to a patient, disease or treatment) to predict some diagnostic or prognostic outcome [1]. Electronic health records store such characteristics in a digital format, thereby facilitating the application of prediction research in clinical practice [7]. Statistical analyses are commonly applied to such data to support individual predictions by population-based evidence. In particular, one branch of statistics is commonly used in this context.

Survival analysis is a collection of statistical procedures for the analysis of data in which the outcome of interest (i.e. survival outcome) is the expected duration of time until an event happens (or, for short, time to event), which is typically referred to as survival time [8]. In the clinical context, survival analysis involves the estimation of the distribution of the time it takes for an event (e.g. death, disease incidence or recurrence) to happen to a patient based on some set of features, which are also known as explanatory variables, predictors or covariates. An important characteristic of survival data is that the follow-up of patients is typically incomplete [1]. For example, some patients may have been followed for 5 days, some for 15 days, etc., yet we may be interested in predicting 30-day survival. Such incomplete data are what we call *censored data*. In essence, censoring occurs when we have some information about individual survival times, but we do not know the survival time exactly in all subjects.

Traditionally the Kaplan-Meier (KM) method, a non-parametric approach, has been used for exploratory analysis of survival data. Using the KM method, survival curves can be generated for various subgroups (e.g. females versus males) to investigate the effects of explanatory variables on survival. However, the KM is unable to consider the effect of multiple explanatory variables simultaneously. To overcome this limitation, several regression modelling approaches have been proposed to enable prediction of time to event in the presence of censored data [9]. Most of these models come from the long-established statistical literature such as parametric survival models, including the Weibull, lognormal and Gompertz models as well as the semi-parametric proportional hazards model proposed by Cox [10]. Hereafter, these will be jointly referred to as linear statistical models. In medical and epidemiological studies, the Cox proportional hazards model (or, for short, Cox regression) is the most prevalently used model for survival outcomes.

Alternative methods for survival analysis are based on machine learning, e.g. artificial neural networks (ANN). Multiple studies have compared such novel non-linear statistical methods with their classic linear counterparts for survival analysis [11–14]. However, the results are mixed as to whether these non-linear methods offer improved performance. For example, Schwarzer et al. [15] reviewed a substantial number of studies that used ANNs in the diagnostic and prognostic classification, concluding that there is no evidence so far that application of ANNs represents real progress in the field of diagnosis and prognosis in oncology. Schwarzer [15] reviewed a number of these comparison studies showing that the majority have claimed equal performance, but could not rule out the possibility of bias. Another important argument against the use of machine learning for survival analysis is that, with few exceptions (e.g. decision tree learning [16]), the learnt relationships between predictors and outcome are typically non-transparent. This makes them difficult to validate by clinicians, which in turn leads to poor adoption in clinical practice.

The main aim of this study is to develop a computational framework for automatically deriving clinical prediction models from survival data. In line with traditional multi-variate statistical modeling [17], the key requirement for an automatically derived model is to allow survival to be assessed with respect to several factors simultaneously as well as offer estimates of the strength of effect for each factor. These requirements imply that the model needs to be explicit and potentially non-linear. Genetic programming (GP) is an evolutionary computation methodology designed to produce such models.

GP is inspired by population genetics and evolution at the population level as well as the Mendelian understanding of the structure and mechanisms [18–20]. It solves complex problems automatically without requiring the user to know or specify the form or structure of the solution in advance [21]. This makes GP well suited to symbolic regression, where, in addition to searching for the solution to the complex associations between predictors and outcome, GP also searches for the optimal model structure. This in turn makes GP well suited to prediction, primarily an estimation problem, where the mutual correlations between predictors and the outcome are to be estimated. GP has been shown to work well for recognition of structures in large data sets and has the intrinsic advantage of automatically selecting features during the evolutionary process [22, 23].

Having no predefined model structure, GP is able to represent complex non-linear associations that could not be achieved using linear regression techniques, and, therefore, may achieve higher predictive accuracy. In fairness, the flexibility of regression models can also be enhanced through the use of fractional polynomials, restricted cubic splines and interaction terms, potentially increasing its predictive accuracy [7, 24–26]. However, this is not normally used in practice as it complicates interpretation of the model. In addition, the correct use of the appropriate regression methods requires extensive statistical knowledge [27].

GP may improve the selection and transformation of predictors, and it may lead to models with good predictive accuracy in new patients [7, 28–31]. Despite its potential, critics state that GP is more prone to over-fitting compared to conventional development methods [29]. On the other hand, GP has got a clear advantage over other machine learning methods in the fact that it produces an explicit "white box" model, thus potentially leading to higher adoption rate in clinical applications.

GP has been used in medical research for classification and, to a lesser extent, prediction. The objective of this study is to assess its value for prediction on censored data for the purposes of survival. This objective is two-fold. Given that GP is a general methodology for the development of mathematical models rather than a specific technique for solving specific problems, we first need to design and implement a GP approach specifically for survival analysis. This entails development of suitable representation of survival data and the methods for evolving survival models as well as evaluating their ‘fitness’. To investigate the practical utility of the GP approach for survival analysis, we compared it against multi-variable Cox regression. Our case study focuses on the development of a clinical prediction model for the occurrence of vascular events in patients with symptomatic cardiovascular disease. We used data from a prospective cohort study (described in Section II) to develop two prediction models, one using Cox regression and the other using GP (both described in Section III). Their performance was evaluated in terms of risk discrimination and calibration. These results are reported in Section IV and discussed in Section V.

## Materials

### Data

This study was carried out using data from the Second Manifestations of ARTerial disease (SMART) study. Details of the ongoing prospective cohort study at the University Medical Centre Utrecht, the Netherlands, designed to identify predictors of future cardiovascular events in patients with symptomatic cardiovascular disease had been described previously [32]. Briefly, we considered a total of 3,873 patients who were enrolled in the study between September 1996 and March 2006. Newly referred patients with a clinical manifestation of atherosclerosis, defined as transient ischaemic attack, ischaemic stroke, peripheral atrial disease, abdominal aortic aneurysm (AAA) or coronary heart disease were enrolled when presenting at hospital. Following an informed consent, patients underwent baseline examination, which involved a standardized vascular screening, including a health questionnaire for clinical information, laboratory assessment, and anthropometric measurements. All cohort members were followed for clinical cardiovascular events for a minimum of three years. During follow-up, patients were asked to fill in a questionnaire on hospitalizations and outpatient clinic visits biannually. When a possible event was reported by a participant, correspondence and relevant data were collected (discharge letters, laboratory radiology results). Based on all information obtained, every event was audited by three physicians from different departments.

The primary outcome was a cardiovascular event, which was defined as cardiovascular death, non-fatal stroke or non-fatal myocardial infarction. Combing predictor events is a common approach in cardiovascular research to increase statistical power [1]. A cardiovascular event occurred in 460 patients during follow-up.

For our study we *a priori* selected 25 candidate predictors based on previous prognostic studies [33, 34]. These 25 candidate predictors included risk factors traditionally associated with future events (hyperhomocysteinemia, intima media thickness (IMT) and creatinine level), demographics (age and sex) and risk factors for vascular events in the general population (smoking, alcohol use, body mass index (BMI), diastolic and systolic blood pressure, lipids and diabetes). Indicators to the location of symptomatic vascular disease (cerebral, coronary, peripheral atrial disease or AAA) and markers of the extent of atherosclerosis (homocysteine, glutamine, creatinine, albumin, IMT and presence of carotid artery stenosis, see Table 1) were also considered as they may be relevant for future events in patients with symptomatic vascular disease. We note that the primary focus of these models is achieving accurate predictions rather than insight into the predictor effects.

**Table 1:** Baseline characteristics of patients in the SMART cohort.

Predictor		Unit	*N*	Test set *N* = 1,291	Training set *N* = 2,582	Test statistic
**Cardiovascular event**			3873	11% (147)	12% (313)	$\chi_{1}^{2}=0.45$, P = 0.51^1^
**Gender: Female**			3873	25% (320)	25% (656)	$\chi_{1}^{2}=0.18$, P = 0.68^1^
**Age years**			3873	52 60 68	52 60 68	F_1,3871_ = 0.03, P = 0.86^2^
**Smoking:**	**Never**		3873	18% (235)	18% (458)	$\chi_{3}^{2}=5.6$, P = 0.13^1^
	**Former**			69% (885)	71% (1826)	
	**Current**			12% (158)	11% (286)	
	**NA**			1% (13)	0% (12)	
**Packyears**		years	3852	5.2 18.2 33.8	6.1 19.5 34.5	F_1,3850_ = 0.79, P = 0.38^2^
**Alcohol:**	**Never**		3873	20% (255)	19% (496)	$\chi_{3}^{2}=1.1$, P = 0.77^1^
	**Former**			11% (141)	10% (267)	
	**Current**			69% (885)	70% (1804)	
	**NA**			1% (10)	1% (15)	
**Body mass index**		Kg/m2	3870	24 26 29	24 26 29	F_1,3868_ = 3, P = 0.084^2^
**Diabetes:**	**0**		3873	76% (983)	78% (2004)	$\chi_{2}^{2}=1.1$, P = 0.59^1^
	**1**			23% (294)	21% (552)	
	**NA**			1% (14)	1% (26)	
**Systolic blood pressure, automatic**		mm Hg	2650	127 140 155	127 139 153	F_1,2648_ = 1.4, P = 0.23^2^
**Diastolic blood pressure, automatic**		mm Hg	2652	73 79 86	73 79 86	F_1,2650_ = 0.01, P = 0.9^2^
**Systolic blood pressure, by hand**		mm Hg	2375	128 140 158	125 139 155	F_1,2373_ = 3.8, P = 0.052^2^
**Diastolic blood pressure, by hand**		mm Hg	2374	75 82 90	74 82 90	F_1,2372_ = 0.2, P = 0.65^2^
**Total cholesterol**		mmol/L	3855	4.4 5.2 5.9	4.3 5.1 5.9	F_1,3853_ = 2.6, P = 0.11^2^
**High-density lipoprotein cholesterol**		mmol/L	3843	0.95 1.15 1.40	0.97 1.18 1.43	F_1,3841_ = 3.8, P = 0.05^2^
**Low-density lipoprotein cholesterol**		mmol/L	3657	2.5 3.1 3.8	2.4 3.0 3.8	F_1,3655_ = 3.2, P = 0.073^2^
**Triglycerides**		mmol/L	3845	1.1 1.6 2.3	1.1 1.5 2.2	F_1,3843_ = 4.1, P = 0.042^2^
**Cerebral**			3873	30% (387)	29% (760)	$\chi_{1}^{2}=0.12$, P = 0.73^1^
**Coronary**			3873	56% (724)	56% (1436)	$\chi_{1}^{2}=0.08$, P = 0.78^1^
**Peripheral**			3873	24% (308)	24% (632)	$\chi_{1}^{2}=0.18$, P = 0.67^1^
**Abdominal aortic aneurysm**			3873	10% (134)	11% (282)	$\chi_{1}^{2}=0.26$, P = 0.61^1^
**Homocysteine**		(μ)mol/L	3410	10 13 16	10 13 16	F_1,3408_ = 2.5, P = 0.11^2^
**Glutamine**		(μ)mol/L	3854	5.3 5.8 6.5	5.3 5.7 6.5	F_1,3852_ = 0.94, P = 0.33^2^
**Creatinine**		mL/min	3856	78 89 102	78 89 101	F_1,3854_ = 0.62, P = 0.43^2^
**Albumin:**	**No**		3873	75% (969)	75% (1928)	$\chi_{3}^{2}=1.1$, P = 0.78^1^
	**Micro**			17% (221)	17% (434)	
	**Macro**			3% (33)	3% (81)	
	**NA**			5% (68)	5% (139)	
**Intima media thickness**		Mm	3775	0.75 0.88 1.05	0.75 0.88 1.07	F_1,3773_ = 0.24, P = 0.63^2^
**Presence of carotid artery stenosis:**	**0**		3873	79% (1020)	79% (2038)	$\chi_{2}^{2}=0.91$, P = 0.63^1^
	**1**			18% (236)	19% (486)	
	**NA**			3% (35)	2% (58)	

Numbers formatted a b c represent the lower quartile, the median, and the upper quartile for continuous variables. *N* is the number of non–missing values. Numbers after the percent sign are frequencies. NA represents missing value. Tests used:

^1^Pearson test

^2^Wilcoxon test

The data set were split randomly into two parts: a derivation set of approximately 66.67% (2,582 patients) and a validation set of approximately 33.33% (1,291 patients). The derivation set was used for model development (both by Cox regression and by GP), whereas the validation set was used to assess the performance of the two models. The aim of both models was to predict the absolute risk of occurrence of a cardiovascular event. Given the availability of follow-up data, 1-, 3-, and 5-year risks could be assessed. At least 10–20 events per candidate predictor were proposed in previous guidelines for the sensible development of predictions models [1, 25, 35, 36]. Therefore, with respect to sample size in the derivation set, the balance of 313 events and 25 predictors was reasonable (see Table 1).

### Missing values

We pre-processed the data in order to deal with the missing values. Multiple imputation is a technique that offers substantial improvements over value replacement approaches based on complete cases or cases matched for age and sex [37]. It involves creating multiple copies of the data and imputing plausible values randomly selected from their predicted distribution. Here, we used multiple imputation to replace missing values for smoking status, packyears, alcohol, BMI, diabetes, systolic blood pressure (SBP), diastolic blood pressure (DBP), total cholesterol (TC), high-density lipoprotein (HDL) cholesterol, low-density lipoprotein (LDL), triglycerides, homocysteine, glutamine, creatinine, albumin, IMT and carotid artery stenosis (see Table 1), generating a total of five imputed data sets. The first set of imputations was used for further analysis (‘single imputation’). Although multiple imputation is preferable from a theoretical view point, single imputation was considered more practical and sufficient to obtain reasonable predictions [1]. Final models were constructed with multiple imputed data sets to check for any relevant differences in point estimates and widening of confidence intervals.

## Methods

### Cox regression

In medical and epidemiological studies, the Cox proportional hazards model (or Cox regression) is the most prevalently used model for survival outcomes [10]. The logistic model is analogous to this model for a binary outcome in uncensored data, where we know whether or not the patient experienced the event in the time horizon of interest. Multi-variable logistic regression model is the most widely used statistical technique nowadays for binary medical outcomes [1, 38, 39]. The Cox regression model is a natural extension of the logistic model in the survival setting [1].

In the derivation set, we fitted a Cox regression model using a modelling strategy similar to the one used for the development of a clinical prediction model on the SMART data set [1]. Briefly, we first fitted a full main effects model. Biologically implausible values were set to missing (prior to imputation) and extreme values truncated at the 1st and 99th centile. To enhance the flexibility of the Cox regression and enable fairer comparison with the (unrestricted) GP, we considered continuous predictors (e.g. age, creatinine, blood pressure) for transformation. Several transformations were considered in adding polynomials, fractional polynomials, transformations (e.g. logarithm, square root, exponential), restricted cubic splines (with varying number of knots) and linear coding (i.e. categorization). To further enhance a fair comparison with GP, we considered interaction effects between predictors.

Key limitations of the Cox regression model include (1) the assumption of proportional hazards where hazard functions in the different strata are proportional over time, (2) the assumption of linearity and additivity which are implicit in regression’s linear combinations, and (3) the fact that the baseline hazard is never specified. All model assumptions relevant to the Cox regression model were tested. A reduced model was obtained by applying a backwards selection procedure with Akaike information criterion (AIC) [40] as the stopping criterion.

Internal validation of the model was performed using a bootstrapping re-sampling procedure [7, 24, 41]. Random samples were drawn (with replacement) from the derivation set with 200 replications, and the backwards selection of predictors for the reduced model repeated each time. Bootstrapping yielded an estimate of optimism of the reduced models as expressed by the concordance statistic, which for a binary outcome is identical to the area under the receiver operating characteristic (ROC) curve. A shrinkage factor was derived from the bootstrap estimates to re-calibrate the model to adjust for optimism. The re-calibrated model was applied to the validation set to estimate its discrimination and calibration on an independent sample. All analyses were carried out in R (v3.0.1) [42].

## Genetic programming

### Basic principles

GP is a search method inspired by the biological model of evolution [7, 43, 44]. It is an extension of the genetic algorithm first described by Holland [45] and Goldberg [46]. In GP, we evolve populations of solutions represented as computer programs. Generation by generation, GP stochastically transforms populations of solutions into new ones [21]. GP finds out how well a solution performs by applying it, and then comparing its behavior to some ideal. This comparison is quantified to give a numeric value called *fitness*. Based on some selection scheme, those solutions (also known as *individuals*) that perform well are chosen to breed and produce new solutions for the next generation. Genetic variation operators, namely *crossover* and *mutation*, are used to create new solutions from existing ones [21]. Unlike genetic algorithms, which only optimize the parameters of the model, GP evolves the actual structure of the approximation model.

**Fig 1:**
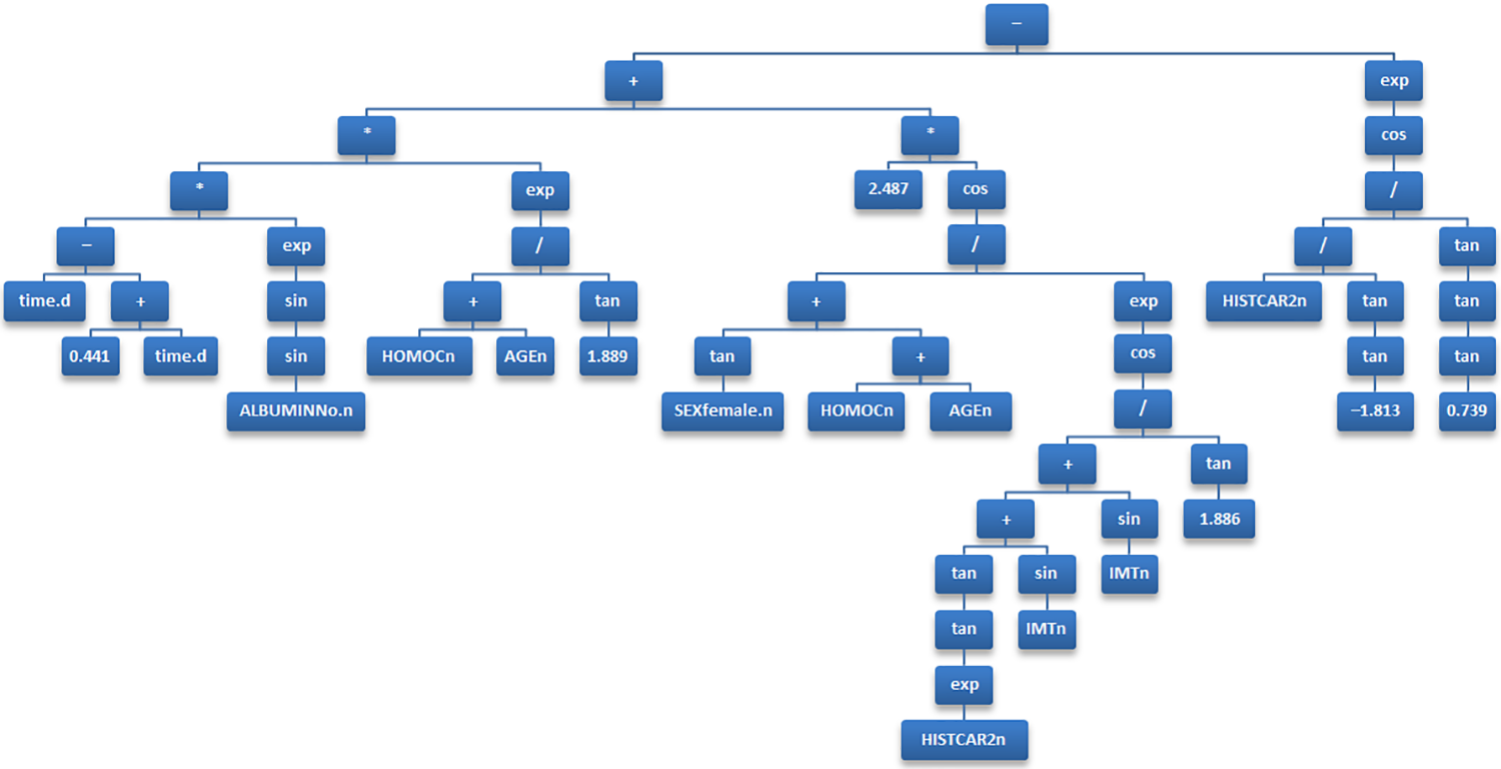
The final model developed by genetic programming, presented as a binary tree.

Various forms of GP differ in the ways in which an individual is represented. The most common form of GP, *tree-based GP*, uses trees as its representation scheme. Consider, for example, a tree shown in Fig 1, which corresponds to a mathematical expression that can be used as a clinical prediction model. The leaves of the tree correspond to input (predictor) variables or constants. The internal nodes correspond to arithmetic operators, which represent the building blocks (or genetic material) that will represent the potential solutions to the problem. Over successive generations, the selection scheme, genetic operators and fitness function will be applied to these building blocks to evolve individuals towards a suitable, hopefully optimal, solution.

### Implementation

For present analysis we used untyped steady-state single-objective tree-based GP [21, 43, 44] to fit symbolic regression models to the data to estimate discrete hazard, thus predicting the risk for cardiovascular events. Here the outcome is discrete hazard rate, which is the conditional probability that a patient will experience the event during time interval [t– Δ/2, t + Δ/2), given they are event-free at the beginning of the interval, as opposed to continuous hazard rate in the Cox regression. An advantage of this model is not constrained by the assumption of proportional hazards and is better suited to any non-linear interactions between explanatory variables. In our implementation of GP, a prediction model is a mathematical formula, without the inherent restrictions in complexity found in regression methods such as Cox regression. All predictors as considered inputs and feature selection are an inherent part of the evolutionary process. The modelling process starts with an initial set of candidate prediction models, which are then iteratively optimized by selecting better models.

In the present study, an initial population of 1,000 different candidate models, i.e. different mathematical formulas using different predictors were randomly created. The fit of all models (to the training data) in the initial candidate set is calculated. Then in an iterative process:

1. A random sample (without replacement) is taken from the current set of models.
2. Genetic variation operators (crossover and mutation) are applied probabilistically to the given subset of models to create new models.
3. The fitness is calculated for all newly created models.
4. The models in the current population are replaced by the newly created models that have better fitness.
5. Steps 1–4 are repeated, until 12 hours of wall (clock) time has passed.
6. The model with the best fit to the holdout data from the final set of models is proposed as the ‘final’ solution.

In each iteration, given two probabilistically selected models, crossover is realized by randomly swapping parts of the model. Mutations occur by randomly replacing part of the model with a randomly generated substitute. A prediction model developed by this type of GP can be presented as a binary tree (see Fig 1 for an example). To limit the amount of optimism, the trees were constrained in terms of complexity by restricting their depth to a maximum of 63 levels. The building blocks of the formulas are arithmetic operators chosen from +, −, /, ×, sin, cos, tan, √, exp, and log. The output of the model is a vector of log odds for each subject and time interval, which is subsequently used to predict the risk of the outcome (i.e. a cardiovascular event).

None of the well-studied fitness measures proved suitable for symbolic regression in the presence of censored survival data. This is because unlike simple linear regression, where these measures would be appropriate, there is no single continuous outcome with which to compare its distance from a models estimate. Instead, in survival problems we have a two-part outcome, with a continuous time until event value and a dichotomous event indicator value. In order to develop a GP fitness function for survival data we take advantage of the fact that the hazard function corresponds to a conditional probability in the discrete time domain. The following equation defines the discrete-time hazard function, which is the conditional probability that the individual *i* will experience the event within time period *j* for the first time given their particular values of the set of covariates, X, in that time period:

$$\hat{h}(t_{ij},X)\;=\;P[T_{i\;=\;j}|T_{i\geq j},X]$$

This is in contrast to hazard in the continuous-time domain, which represents a rate, and as such can take values greater than one. The corresponding discrete-time survival function is defined as follows:

$$\hat{S}(t_{ij},X)\;=\;\prod_{k\;=\;1}^{j}\hat{h}(t_{ij},X)$$

It follows that the original survival analysis problem can be cast into a classification problem that requires the estimation of a conditional probability. However, to address the problem of censoring, the data needs to be converted into the ‘counting process format’, in which there are multiple rows per subject, one for each observed discrete-time interval. An example of survival data in this format is given in Table 2, where *X* is a set of *P* covariates *x*_1_, …, *x*_*P*_. One advantage of the ‘counting process format’, that it can inherently represent a combination of time-varying covariates (e.g. cholesterol) as well as static covariates (e.g. gender).

**Table 2:** Example of survival data in the counting process format.

Patient	Time	Event	*x*_1_	…	*x*_*P*_
1	1	0	1	…	0
1	2	0	1	…	1
2	1	0	0	…	1
2	2	0	0	…	0
2	3	1	0	…	1

We can now reformulate the discrete-time hazard function as the conditional probability P(EVENT|*X’*), where *X’* is a vector consisting of the original covariates found in X together with an additional time period indicator *j*. This probability can be estimated using the likelihood and prior ratios with a logistic link function:

$$h(t_{ij})\;=\;\frac{1}{1+e^{-\varepsilon }}$$

In the case where ε is a linear combination of covariates X’ (including time indicator *j*), this represents a logistic regression model, which can be optimized using standard statistical techniques such as Newton-Raphson method. The following equation defines *ε*_*lp*_ as a linear predictor for discrete time survival analysis:

$$\varepsilon_{lp}\;=\;[\alpha_{1}D_{1ij}+\alpha_{2}D_{2ij}+\cdots +\alpha_{J}D_{Jij}]+[\beta_{1}X_{1ij}+\beta_{2}X_{2ij}+\cdots +\beta_{P}X_{Pij}]$$

In this definition *D*_*ij*_ is a ‘dummy’ time indicator, a dichotomy whose value indexes the time period *j* in the *i-*th individual, *P* is the number of predictors (or covariates) and *J* is the number of observed time periods.

However, if we adopt a more complicated relationship for ε using a symbolic expression, we can model a non-linear relationship between hazard and covariates. It can be optimized by GP search operators, using the following likelihood function:

$$\prod_{i\;=\;1}^{n}\prod_{j\;=\;1}^{J_{i}}{h(t_{ij})}^{{EVENT}_{ij}}{\left(1-h(t_{ij})\right)}^{1-{EVENT}_{ij}}$$

Here, EVENT_*ij*_ is a dichotomy representing the event indicator of the *i*-th individual at the *j*-th time interval. We define *n* as the number of subjects in training data and *J*_*i*_ as the number of observed time periods the *i*-th individual contributes to the likelihood function. To make optimization through the GP search operators more computationally tractable, we take the logarithm of the likelihood to form a fitness function for survival analysis in censored data:

$$-\sum_{i\;=\;1}^{n}\sum_{j\;=\;1}^{J_{i}}\left({EVENT}_{ij}\text{log}\;h(t_{ij})+(1-{EVENT}_{ij})\text{log}\;\left(1-h(t_{ij})\right)\right)$$

The fitness function expresses the joint probability of obtaining the data actually observed on the subjects in the study as a function of the unknown population parameters.

### Validation

To understand variable selection in the GP and enable comparison with bootstrapped backwards selection of the Cox model, the GP system was executed 25 times to produce a set of 25 models. For each iteration, the training data were randomly (stratified) split in a 2:1 ratio. The final GP model was applied to the validation data set to assess its performance in terms of discrimination and calibration on an independent sample.

We did not perform internal validation in the GP approach using a bootstrap as we did with the Cox regression, because it would not have been possible to convert it into a shrinkage factor in the same way as we would for a Cox regression. The GP system is a stochastic process, with each run potentially yielding models with differing complex structures (i.e. symbolic regression). As a result, regression coefficients do not exist in GP models in the same way that they do in regression models. Instead the training data was split 2/3:1/3 into training and holdout sets, using a stratified random split to ensure proportionate number of events. The first 2/3, the training set, was used for training to induce a population of prediction models. The remaining 1/3, the holdout set, was used at the end of the GP run to calculate the fitness of the population of models and thus determine the fittest or ‘best of run’ model to be returned as the output of the GP system. In this way the final GP model was selected based on its fit to unseen data using a sample other than the one it was trained on. All analyses were carried out in R version 3.1.2 [42].

## Results

### Evaluation measures

The two clinical prediction models obtained from Cox regression and from GP, were evaluated in terms of overall survival curves, discrimination and calibration in the validation data set. The models were used to predict the discrete hazards *h*(*t*) at *t* = 1, 3, and 5 years. The models were first evaluated by comparing the survival probabilities *S*(*t*) predicted by the models against estimates obtained using the KM method. The agreement between these curves and the KM estimates were assessed visually.

Discrimination is the ability of the risk score to differentiate between patients who did and did not experience an event during the study period. This measure was quantified by calculating a concordance statistic (or C-statistic) proposed by Harrell et al. [24, 47–49], which is a rank-based measure for censored survival data. The C-statistic is an equivalent of the area under the curve (AUC) measure [38] for survival data, in which 0.5 represents random chance and 1 represents perfect discrimination. The C-statistic was evaluated considering truncation of the survival/censoring times at *t* = 1, 3, and 5 years.

In the context of this study, calibration refers to how closely the predicted *x*-year cardiovascular risk agrees with the observed *x*-year cardiovascular risk. Model calibration was assessed using calibration plots and the generalization of the Hosmer-Lemeshow test statistic for survival data [50]. This was assessed by grouping subjects into *g* equally sized groups, with the same cardinality, based on quantiles of predicted *S*(*t*), where *t* is a fixed time point, and calculating the ratio of predicted to observed cardiovascular risk. For each of the *g* groups, plotting observed proportions (KM estimates) versus predicted probabilities (by the model) enabled the calibration of the model predictions to be assessed visually. The closer the *g* points to the 45 degree line connecting (0,0) to (1,1), the better the calibration. To obtain the χ^2^ statistic, the model predicted number of events was calculated, for each group, as the product of the group size by the average predicted incidence 1 − *S*(*t*). The results were then compared to the observed number of events in the corresponding groups calculated as the product of the group size by the KM estimate of 1 − *S*(*t*). This leads to a statistic which, under the null hypothesis of numerical agreement between predicted and observed number of deaths, has a χ^2^ distribution. Calibration was evaluated by grouping subjects according to the predicted values of *S*(*t*) at *t* = 1, 3, and 5 years. All analyses were carried out in R version 3.1.2 [42].

### Descriptives

There were no major differences in the baseline characteristics of the patients between the derivation and validation sets (see Table 1). Data were available on 9,636 and 4,895 person-years collected during a median follow-up of 3.3 (0–9 years) and 3.3 years (0–9 years) for the derivation and validation sets respectively. In the derivation set, a total of 313 events occurred, corresponding to 1-, 3-, and 5-year cumulative incidences of 4.1% (108), 8.9% (80), and 15.0% (84) respectively. In the validation set, a total of 147 events occurred, corresponding to 1-, 3-, and 5-year cumulative incidences of 3.8% (50), 8.1% (36), and 12.0% (33) respectively.

### Model derivation

Prior to modelling extreme values in IMT, BMI, lipids (cholesterol, HDL, LDL, triglycerides), homocysteine and creatinine were truncated at the 1st and 99th centile. Indicators to the location of symptomatic vascular disease (cerebral, coronary, peripheral atrial disease or AAA) were optimally combined into a single variable (or sumscore), with each condition contributing one point except AAA that contributed 2 points. Using univariate Cox models, no significant difference were found in the sumscore (χ^2^ 119; 1 degree of freedom) using the separate terms (χ^2^ 123; 4 degrees of freedom). However, there was a saving of 3 degrees of freedom from the sumscore.

The full Cox regression model consisted of 14 predictors, several of which had limited contributions. Predictors that had a relatively large effect include age, location of symptomatic vascular disease (sumscore), albumin, and creatinine. The coding of predictors that gave the best representation for age and creatinine were (AGE– 50)^2^ and log(CREAT) respectively. We also tested interactions between predictors, but the resultant interactions were not considered relevant enough to include any interaction terms in the final model. The proportionality of hazards was tested using an overall test which was not significant. We judged our sample size to be large enough to allow for some model reduction (313 events and a full model with 17 degrees of freedom), facilitating easier practical application and clinical interpretation. We applied a backwards step-wise selection procedure using AIC as the stopping rule to obtain a reduced Cox model. The reduced step-wise selected model was found to be optimal with 9 predictors (see Table 3). Predictors with relatively weaker effects (alcohol, diabetes, gender, smoking status, and stenosis) were excluded from the reduced model. Bootstrapping of the reduced model yielded an estimate of required shrinkage for the coefficients in the step-wise selected model of 0.91, suggesting that each coefficient should be reduced by 9% to obtain a re-calibrated model that corrects for optimism. This shrinkage factor was applied to the reduced backwards step-wise model and considered the calibrated ‘final’ Cox regression model (see Table 4). All analyses were repeated with the multiple imputed data sets with similar results.

**Table 3:** Cox regression coefficients.

Predictor	Variable	Full	Stepwise
**Age**	AGE	0.0011	0.0011
**Albumin**	ALBUMIN = Macro	0.5289	0.5371
	ALBUMIN = Micro	0.5227	0.5184
**Alcohol**	ALCOHOL = Current	0.0234	
	ALCOHOL = Former	−0.1854	
**Body mass index**	BMI	−0.0383	−0.0359
**Creatinine**	CREAT	0.5992	0.5282
**Diabetes**	DIABETES		0.0783
**High-density lipoprotein cholesterol**	HDL	−0.4619	−0.4096
**Previous atherosclerosis (sum score)**	HISTCAR2	0.2980	0.2895
**Homocysteine**	HOMOC	0.0169	0.0182
**Intima media thickness**	IMT	0.5145	0.5879
**Gender**	SEX = Female	0.1754	
**Smoking**	SMOKING = Current	0.0798	
	SMOKING = Former	0.0427	
**Presence of carotid artery stenosis**	STENOSIS	0.1815	
**Systolic, by hand**	SYSTH	0.0037	0.0041

**Table 4:** Association of predictors with cardiovascular events.

	Low	High	Δ	Effect	S.E.	Lower 0.95	Upper 0.95
**AGE**	52.00	68.0	16.00	0.32	0.08	0.16	0.48
Hazard ratio	52.00	68.0	16.00	1.38		1.18	1.61
**BMI**	24.03	28.7	4.69	–0.15	0.08	–0.31	0.00
Hazard ratio	24.03	28.7	4.69	0.86		0.73	1.00
**SYSTH**	127.00	156.0	29.00	0.11	0.07	–0.04	0.25
Hazard ratio	127.00	156.0	29.00	1.11		0.96	1.29
**HDL**	0.96	1.4	0.47	–0.18	0.09	–0.34	–0.01
Hazard ratio	0.96	1.4	0.47	0.84		0.71	0.99
**HISTCAR2**	1.00	5.0	4.00	1.05	0.27	0.52	1.59
Hazard ratio	1.00	5.0	4.00	2.87		1.67	4.91
**HOMOC**	10.50	15.9	5.40	0.09	0.05	–0.02	0.19
Hazard ratio	10.50	15.9	5.40	1.09		0.98	1.21
**CREAT**	78.00	101.0	23.00	0.12	0.05	0.04	0.21
Hazard ratio	78.00	101.0	23.00	1.13		1.04	1.24
**IMT**	0.75	1.1	0.32	0.17	0.07	0.04	0.30
Hazard ratio	0.75	1.1	0.32	1.19		1.04	1.35
**ALBUMIN—Micro:No**	1.00	2.0		0.47	0.14	0.21	0.74
Hazard ratio	1.00	2.0		1.60		1.23	2.09
**ALBUMIN—Macro:No**	1.00	3.0		0.49	0.24	0.02	0.96
Hazard ratio	1.00	3.0		1.63		1.02	2.61

The calibrated final Cox model.

The final model produced by the GP model included 6 predictors: age (AGEn), sumscore of previous atherosclerosis (HISTCAR2n), gender (SEXfemale.n), IMT (IMTn), homocysteine (HOMOCn), and albumin (ALBUMINNo.n), in addition to the discrete time indicator (*t*_*j*_), which was present in all GP models to represent the *j-*th time interval. The final prediction model generated by GP is presented in Fig 1, which is a binary parse tree representing the following equation:

$$\hat{\lambda }(t_{j},X)\;=\;Prob(T\;=\;t_{j}T\geq t_{j},X)\;=\;\frac{1}{1+e^{-X\hat{\beta }}},$$

where

$$\begin{array}{l} X\hat{\beta }=\left(t_{j}-\left(0.441+t_{j}\right)\right)\cdot \text{exp}\left(\text{sin}\left(\text{sin}\left(\text{ALBUMINNo.n}\right)\right)\right)\cdot \text{exp}\left(\left(\text{HOMOCn}+\text{AGEn}\right)/\text{tan}\left(1.889\right)\right)\\ \begin{array}{l} \cdot \text{cos}(\left(\text{tan}\left(\text{SEXfemale.n}\right)+\text{HOMOCn}+\text{AGEn}\right)/\text{exp(cos}((\text{tan}\left(\text{tan}\left(\text{exp}\left(\text{HISTCAR2n}\right)\right)\right)+\text{sin}\left(\text{IMTn}\right)\\ +\text{sin}\left(\text{IMTn}\right))/\text{tan}\left(1.886\right))))\cdot 2.487 \end{array}\\ -\text{exp}\left(\text{cos}\left(\text{HISTCAR2n}/\text{tan}\left(\text{tan}\left(-1.813\right)\right)/\text{tan}\left(\text{tan}\left(\text{tan}\left(0.739\right)\right)\right)\right)\right) \end{array}$$

The GP approach was applied 25 times, each time trained and tested on a different stratified re-sample of the derivation data set. This leads to a pool of 25 different ‘best of run’ models, each of which may have selected different subset of predictors as inputs and as such may have differing levels of performance. In this pool of GP models, the mean number of predictors used was 6, with interquartile range (IQR) 5–8. The backwards step-wise selection procedure used in the Cox modelling was also repeated 25 times, using bootstrap re-sampling to better understand the frequencies at which different subsets of predictors were selected. In the pool of 25 backwards selected Cox models, the mean number of predictors used was 9 (IQR 8–10). There was a reasonable association between the estimated effect of a predictor according in the reduced backwards step-wise model and the frequency of the selection when the step-wise selection was repeated in the bootstrap procedure.

#### D. MODEL VALIDATION

**Fig 2:**
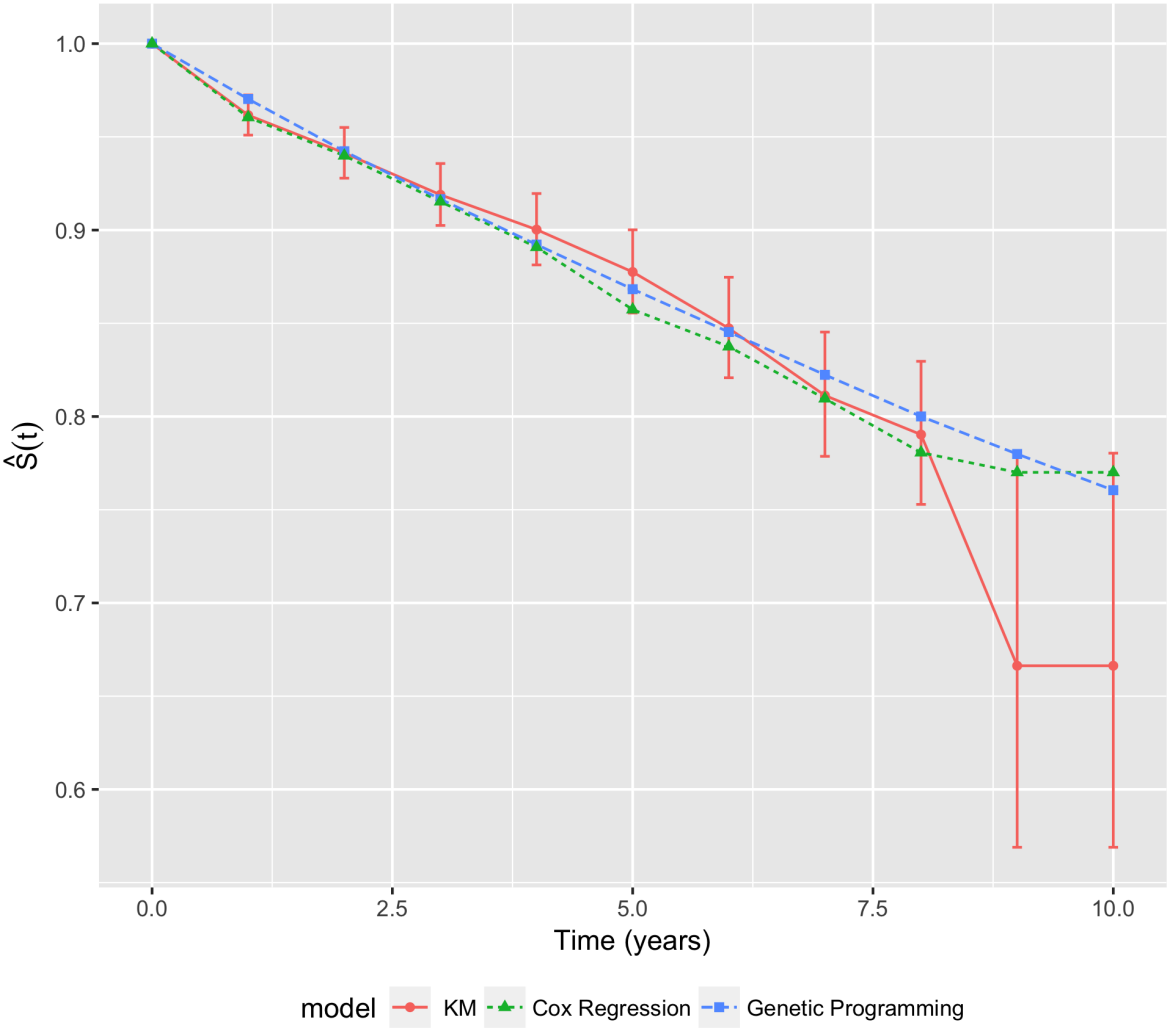
Average survival curves for the Cox regression and GP models. The error bars represent ±2 standard errors of the KM estimates.

Using the validation data set, the average performance of the 25 ‘best of run’ prediction models automatically generated by GP was compared with the calibrated final Cox model. Graphical comparisons of the *S*(*t*) values produced by each model with those obtained by the KM method in the validation set are shown in Fig 2. Both Cox and GP models produced similar values that had good agreement with the KM estimates in the earlier years. However, this agreement deteriorated in the latter years, where the KM estimates have high variability as indicated by the large error bars. This high variation may be explained by the fact that, with a median follow-up time of 3.3 years, there were far fewer events and subjects in the latter time periods. Whilst the agreement deteriorated in the latter time-points, both models had generally acceptable overall agreement.

**Fig 3:**
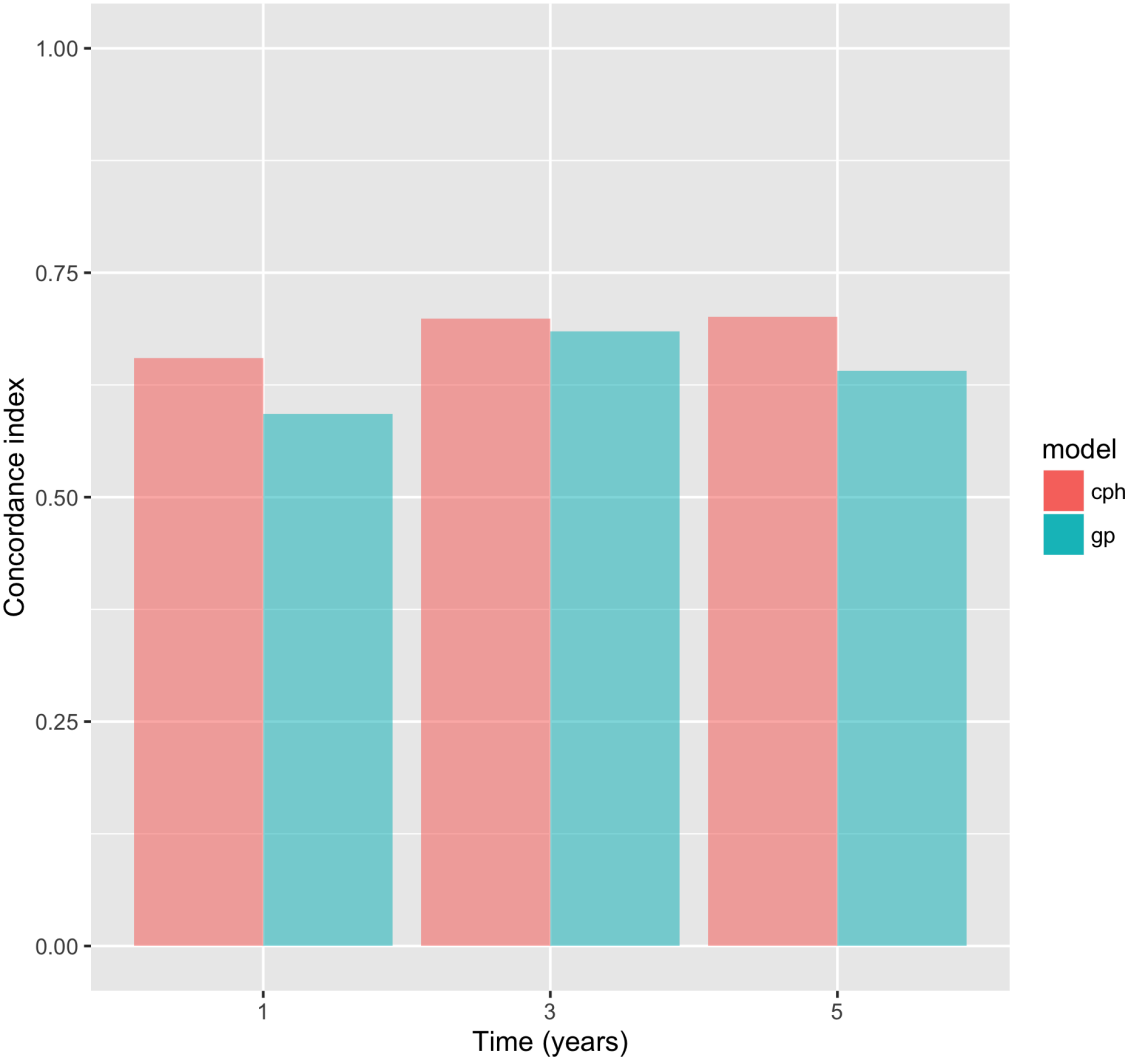
C-statistic estimates by model for *t* = 1, 3 and 5 years.

The discriminative performance in the validation set, according to the C-statistic, of the models at different time points is shown in Table 5 and Fig 3. The lowest estimate of the C-statistic was approximately 0.6, which implies that a satisfactory performance was observed in both models at all time points. There was generally comparable discriminative performance of both models at all time points, albeit in favor of the Cox model. Both models demonstrated better performance at time *t* = 3 years, which may be explained by the 3.3 median follow-up time in the validation set.

**Table 5:** C-statistic.

Time (years)	Cox regression	GP
**1**	0.66	0.59
**3**	0.70	0.69
**5**	0.70	0.64

Values estimates by the two models at *t* = 1, 3 and 5 years.

**Fig 4:**
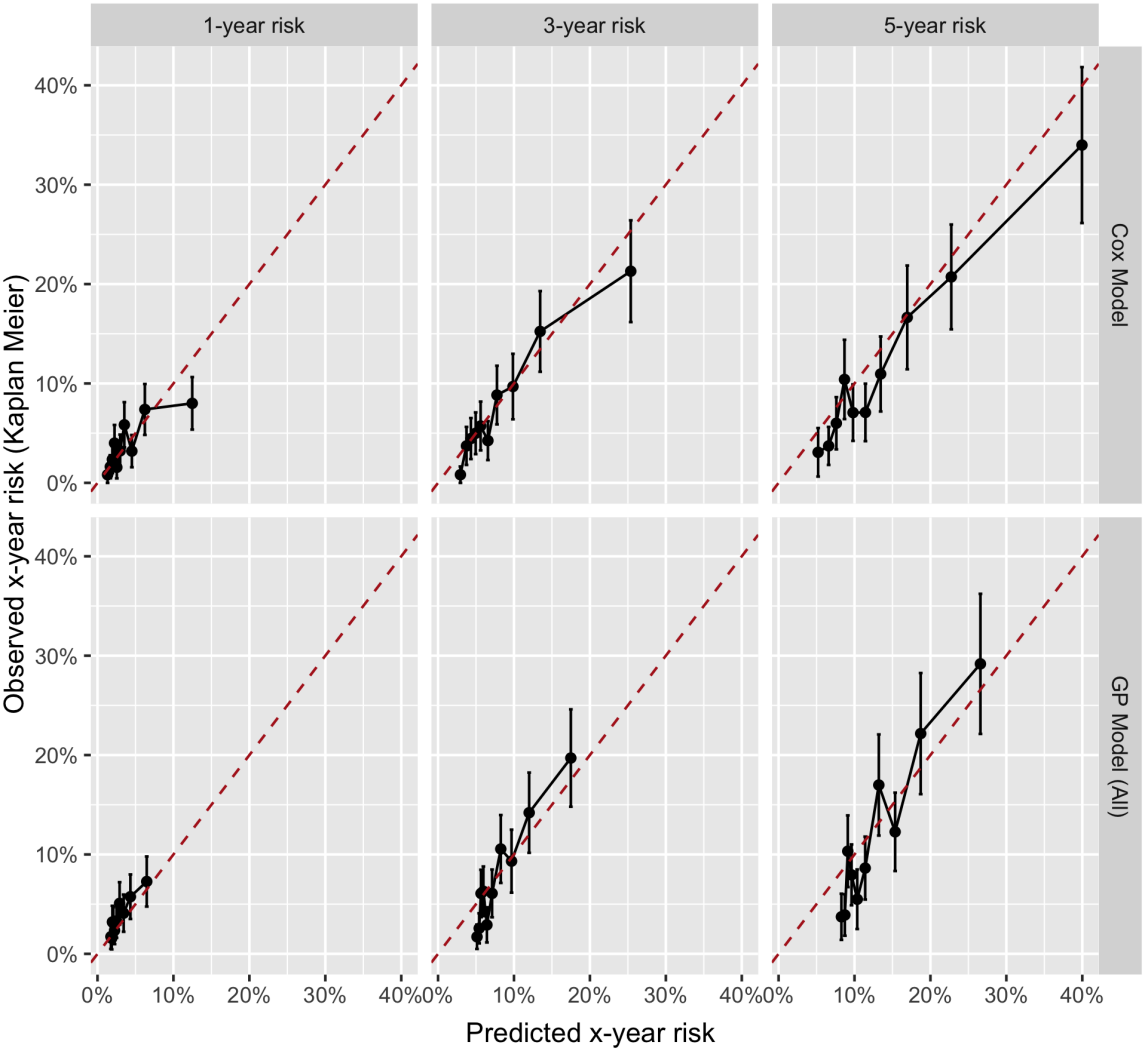
Calibration plots for the Cox regression and GP models at *t* = 1, 3 and 5 years.

The calibration plots evaluated by grouping subjects according to quantiles of predicted risk 1 − *S*(*t*) at *t* = 1, 3, and 5 years are shown in Fig 4. The corresponding χ^2^ statistics and *p*-values are shown in Table 6. From the graphical inspection of the calibration plots, we can see that there was no tendency to systematically over- or under-predict at any of the time points in either Cox or GP models. The GP model was less calibrated than the Cox model, confirmed by the higher χ^2^ values in Table 6 at times *t* = 3 and *t* = 5, whereas it was better calibrated at time *t* = 1. Calibration in both models was worst at time *t* = 5, and best in the Cox and GP models at times *t* = 3 and *t* = 1 respectively. However, the Homser-Lemeshow test statistic, detailed in Table 6, suggested that there was only a statistically significant lack of calibration in the GP model at time point *t* = 5.

**Table 6:** χ^2^ statistic.

Time	Cox regression		GP	
(years)	χ^2^	p-value	χ^2^	p-value
**1**	7.93	0.541	5.18	0.818
**3**	4.89	0.844	9.99	0.352
**5**	10.32	0.325	16.17	0.063

A comparison between observed and expected (according to the model) number of events in groups of patients defined according to the predicted 1 − *S*(*t*) at *t* = 1, 3 and 5 years.

## Discussion

This study demonstrated that Cox regression and GP produced comparable results when evaluated on a common validation data set. After re-calibration, the discriminative ability of the GP on the validation set was slightly larger than that of the Cox model at two time points, whereas the Cox model was marginally better at only one time point. Despite slight relative differences, both models demonstrated an acceptable level of discriminative ability (C-index approximately 0.6 or higher) at all times points. The GP model had relatively poorer calibration when compared with the Cox model. The Cox model demonstrated no significant lack of calibration at any time point. However, the GP model did demonstrate a significant lack of calibration at the latter time point only.

### Selection

Despite generally comparable performance, albeit in favor of the Cox model, the predictors selected for representing their relationship with the outcome were quite different. The final reduced Cox model used 9 predictors, in contrast to 6 predictors used in the GP model. The GP model used fewer predictors, further confirmed by repeating the GP and the stepwise selection procedure used in the Cox modelling, resulting in mean numbers of predictors of 6 (IQR 5–8) and 9 (IQR 8–10) respectively. This finding suggests that GP may be better at representing the potentially non-linear relationship of (a smaller subset of) the strongest predictors. Whilst considerable effort was made to relax the linearity of the Cox regression through transformation of predictors, the nature of the approach relies on linear combinations of predictors. The fact that GP required fewer predictors to achieve similar performance may have an advantage in practical application of the developed clinical prediction model. The acquisition of information that forms the inputs to such a model can be prohibitively onerous in routine clinical practice. Therefore, a prediction model that requires fewer inputs, especially if the information relating to these inputs is in practice recorded easily and to a good quality, would considerably increase adoption and utility.

### Interpretation

Unlike many other machine learning algorithms, GP in not a ‘black box’ method and provides an explicit mathematical formula as its output. However, the model structure in the GP model is typically more complex than that of the Cox regression model. This hinders the interpretation of the relative effects of predictors on the outcome. If the primary objective of the modelling is to understand these effects, such as in aetiologic research, then Cox regression and other related approaches still remain the first choice. However, if the primary goal of the research is an accurate risk prediction, then GP has some utility when compared with its regression counterpart. GP offers advantages that include the ability to learn complex non-linear relationships that may exist within the data. It is not constrained by the statistical assumptions that underpin Cox regression (such as proportional hazards). On the contrary, GP has inherent feature selection and develops models in a fully automated fashion.

### Optimism

An advantage of Cox regression is its ability to be calibrated using all data available by applying a shrinkage factor, a measure of the model’s optimism (or over-fitting) estimated though bootstrapping or penalized regression methods. On the other side, GP cannot estimate a shrinkage factor in the same way and needs a validation sample. This suggests that in cases where the data are scarce, Cox regression may be a better approach. By contrast, in cases where there the data are abundant, possibly with a large number of predictors and potential interactions, GP would have a distinct advantage. Whilst interaction effects can be modelled using regression techniques, this can be onerous and requires significant statistical expertise.

### Expertise

The considerable statistical and clinical expertise required in the development of appropriate clinical predictions should not be understated. Problems with step-wise feature selection methods are another concern; including biased *R*^2^ values, confidence intervals for effects and predicted values that are falsely narrow, biased regression coefficients that need shrinkage, and severe problems in the presence of collinearity. Both Cox regression and step-wise selection are widely used and widely abused in prognostic and aetiologic research. Whilst fitting models using these techniques is relatively straightforward and intuitive, sometimes they are applied blindly without appropriate testing of the underlying assumptions. Cox regression is a powerful tool, but its correct application requires a certain amount of statistical rigor and expertise from the researcher, and cannot be used with certain data if its underpinning assumptions are violated. Another weakness of a Cox model is that it does not explicitly define the underlying baseline hazard, which means that, technically, its predictions are only valid at the time points observed in the data and that it may not appropriate for extrapolation to non-observed time points. It should be noted, however, that other regression methods for survival analysis, such as parametric survival models, can define the baseline hazard and are appropriate for extrapolation. However parametric modelling of survival is even more involved than Cox modelling, requiring greater technical expertise, and as such features far less in published aetiologic research.

### Efficiency

The main weakness of the GP approach is that the data need to converted into the counting process format (i.e. time-coded), which leads to large data sets and long executions times. Whilst in theory GP works better on large data sets, the long execution times can make its use prohibitive in practice. However, this weakness can be addressed though parallel processing. GP is a method that can be described as naturally parallelizable, and as such can be adapted to execute in parallel across multiple machines or processors.

### Clinical context

This work has limitations introduced by its use of data from the SMART study, a study from a secondary prevention setting, designed to predict the risk of subsequent cardiovascular events in patients with already presenting with clinical cardiovascular disease. Through the use of the SMART study data, this work has demonstrated the utility of GP in a secondary prevention setting. However, there are limitations in the generalizability of these findings to other clinical settings of cardiovascular risk prediction. Indeed, secondary prevention in stable cardiovascular patients is not the most common clinical setting for the application of cardiovascular risk prediction models in routine practice. Further work is required to assess the utility of GP for automated development of new clinical prediction models in other clinical and environmental settings, preferably comparing their performance against more established risk prediction models currently used in routine clinical practice, e.g. QRISK 2 [51] and SCORE [52] for primary prevention of CVD, the GRACE score [53, 54] in acute coronary syndromes and, euroSCORE [33] in cardiac surgery. Further work will also be required to identify the most appropriate clinical parameters required for GP risk modelling in order to optimize the predictive power in the relevant setting and to ensure that these required measures are not only practical to measure at scale in routine clinical practice but also cost effective.

### Parameters

Finally, the GP model has a number of parameters that need to be specified *a priori*. These parameters include the size of the population of models, the building block of models such as mathematical operators, the number of runs to perform, the rates at which to apply genetic variation, and parameters such as maximum tree depth that control the complexity, and thus potential of over-fitting of the final GP model. Often, the choice of these parameters is based on trial and error, model tuning or existing literature. Model tuning refers to repeating the same experiment many times whilst simultaneously varying multiple parameters and quantifying relationship between them and the quality of resultant models to understand which parameters are important.

However, little or no literature discusses the relative importance of specific parameters of survival analysis. Model tuning was outside of the scope of this study, but further research is warranted into characterizing the association of GP parameters and performance in a survival analysis setting. Our choice of starting parameters was driven by the size of the training data available, the perceived relative complexity of the problem and, the recommendation and guidelines from the relevant literature. Wherever possible we opted for the most common or default operators and parameter settings, opting not to tune the parameters. The default set of operators (namely, +, −, ÷, ×, sin, cos, tan, √,exp, log) was used to enable the representation of potential non-linear relationships present in the training data. Koza’s [44] ramped half-and-half random initialization method, the most commonly used initialization operator in tree-based GP. Some authors propose that, as a rule of thumb, to specify the maximum tree depth, one should try to estimate the size of the expected solution size and add some percentage as a safety margin. For this experiments we calculated, when transforming categorical predictors into ‘dummy’ variables (i.e. *n* − 1 dummies per categorical predictors, where *n* is the number of levels), that the expected solution depth would be 21 based on the expected solution being modelled as a regression model with 19 predictors. Based on this we estimated a maximum depth should be 21 × 3% = 63. In most real-world GP applications only a fixed compute time budget is available. Therefore, the expiration of a fixed time compute budget was chosen as the termination criterion. The compute time budget for these experiments was set to 12 hours wall-time and these experiments were run on a single thread on an Intel Westmere 2.8GHz CPU with 48GB of memory. Based on observations from GP practitioners, we opted for a relatively large population size of μ = 1000, which tends to have suitable genetic diversity. The most commonly used mutation and recombination operators in tree-based GP, subtree mutation and subtree crossover were selected. Because of relatively high complexity of the problem at hand, we set a relatively high genetic variation rate of 0.5 for both mutation and crossover.

## Conclusions

The main aim of this study was to develop a computational framework for automatically deriving clinical prediction models from survival data. For this purpose, we used GP, an evolutionary computation methodology designed to derive explicit mathematical models from large datasets. An obvious advantage of this approach in comparison with traditional machine learning methods is the transparency of learnt relationships between predictors and outcome. This makes it easier to be understood and validated by clinicians, thus improving the adoption of machine learning approaches in clinical practice. Unlike multivariate statistical modelling, which is routinely used in survival analysis, GP does not require the expertise (both clinical and statistical) normally needed for model derivation, thereby alleviating the knowledge elicitation bottleneck. Having considered these advantages of GP, its suitability for survival analysis depends crucially on the predictive ability of its survival models. Using empirical data, we demonstrated that a model developed automatically by GP has the predictive ability comparable to that of manually tuned Cox regression. However, in terms of practical utility of the GP approach for survival analysis, other properties of the two respective models need to be considered as well. For example, the complexity of the GP-derived model was found to be much higher. While higher complexity may lead to higher accuracy, it may also reduce the transparency of a model by convoluting the effects of and relationships between individual predictors. Fortunately, the complexity consideration can be embedded into the fitness function, and, therefore, fully controlled.

To our knowledge, this is the first empirical study to assess the value of GP for clinical prediction purposes compared to the well-known and widely applied Cox regression technique. Whilst the highly tuned Cox regression model performed marginally better on the validation data, both in term of calibration and discrimination, the performance of the automatically generated prediction model was generally comparable. The comparable performance demonstrates the utility of GP for clinical prediction modelling and prognostic research, where the primary goal is accurate prediction. In etiological research, where the primary goal is to examine the relative strength of association between risk factors and the outcome, Cox regression (and its variants) remains the *de facto* approach.

Further work is required to characterize relationship between GP parameters and the performance of the resulting survival models, as is further research into reducing the execution times through parallel processing. Finally, further validation is required to assess the utility of GP for automated development of new clinical prediction models in other clinical and environmental settings.
